# Reintroducing Guanaco in the Upper Belt of Central Argentina: Using Population Viability Analysis to Evaluate Extinction Risk and Management Priorities

**DOI:** 10.1371/journal.pone.0164806

**Published:** 2016-10-14

**Authors:** Fernando Rafael Barri

**Affiliations:** Instituto de Diversidad y Ecología Animal (IDEA), CONICET-UNC and Facultad de Ciencias Exactas Físicas y Naturales, Universidad Nacional de Córdoba, Av. Vélez Sarsfield 299, CP 5000, Córdoba, Argentina; Université de Sherbrooke, CANADA

## Abstract

Wildlife reintroduction is an increasingly used strategy to reverse anthropocene defaunation. For the purpose of ecosystem restoration, in 2007 the guanaco (*Lama guanicoe*) was reintroduced to the Quebrada del Condorito National Park, situated in the mountains of central Argentina. With the aim of developing management recommendations, the project included permanently monitoring the population to evaluate its dynamics and the ecological response of the individuals released into the area. Nine years later and after two releases of guanacos (113 individuals in 2007 without and 25 in 2011 with a pre-adaptation period), only 24 individuals, which conform three reproductive groups, and one group of solitary males were settled in the Park. Here I modeled a population viability analysis to evaluate extinction risk, using VORTEX software. Initial population structure, specified age distribution, mortality and reproductive rates, and mate monopolization recorded during field work were used in the model, whereas the remaining used demographic parameters, such as age of first offspring, maximum number of broods per year, mean foaling rate, and length of fecundity period, were taken from the literature. Each of the three different scenarios (without supplementation of individuals, and with a realistic and optimistic supplementation) and two possible catastrophic events (fires and food shortage) covering 100 years was repeated 1000 times. Even though the guanaco reintroduction project can be considered to have been partially successful since its start, the model predicts that the current reintroduced population could be extinct in the next few decades if no reinforcements occur, and that only a continuous supplementation can reach the probability that the population survives over the next 100 years. I conclude that, so far, the current population is at a high risk of extinction if further supplementation of individuals is discontinued.

## Introduction

We are experiencing anthropocene defaunation [1], which implies massive biodiversity extinction during the last century and declines of current wildlife population abundance [2]. Refaunation involves re-establishing species into areas or ecosystems where they became locally extinct [3]. Accordingly, wildlife reintroduction has notably increased during the last decades [4], [5]. Reintroductions are suggested when they favour ecosystem functionality and are conducted over a long period, with emphasis on restoring natural processes rather than addressing only extinction risk [6]. However, reintroductions are complex and costly processes that may fail due to a lack of knowledge on the ecology of the species in the habitats where they are reintroduced [7]. Hence, it is highly recommended that reintroduction projects include continuous monitoring [8], because the obtained information helps to make proper management decisions [9].

Population viability analysis (PVA) models have been used to evaluate extinction risks, population trends, and management priorities for natural and reintroduced populations [10–14]. Particularly in reintroductions, the model should project population size, population growth rate and probability of extinction some years after releases ended [15]. Moreover, explicitly modelling the post-release effects is useful for guiding decisions about the optimal number of individuals to be released, the necessary period length (years) of release and the most appropriate release methods [16]. Therefore, population modelling is critical for managing reintroduction efforts and helps to increase the chances of effective species recovery around the world [4].

The guanaco (*Lama guanicoe*) reintroduction project in the upper belt of the mountains in central Argentina was developed to recover a large native herbivore that historically inhabited the region. In 1996 the National Parks Administration (NPA) created the Quebrada del Condorito National Park (QCNP) to protect a fragile rangeland ecosystem. Since the Spanish colonization, this area had suffered a widespread process of soil erosion and replacement of the vegetated surface with exposed bedrock due to cattle overgrazing and the frequent use of fire to induce grass regrowth [17], [18]. This area is important for water supply to millions of people [19]. Even though domestic livestock were removed from a large area of the QCNP, their exclusion caused a disproportionate expansion of a thick-leaved tussock grass at the expense of grazing lawns, reducing local diversity and spatial heterogeneity [20], [21]. In an attempt to control landscape homogenisation and at the same time avoid soil erosion processes induced by livestock, in 2007 the guanaco, a low-impact grazer, locally extinct early in the 19th century by intensive hunting [22], [23] was reintroduced.

The reintroduction of guanacos involved a continuous monitoring of the population, because the success or the causes of failure can be assessed only through adequate post-release monitoring [24]. In addition, different studies conducted during the last years evaluated the adaptation process of the reintroduced guanacos to the area. For example, reintroduced guanacos that survive the first three months of the critical post-release period respond adequately in terms of social group behaviour [25]; they positively selected grazing lawns from the different habitats available in the area [26]; and their diet consisted mostly of a group of palatable species of these grazing lawns [27]. Although the obtained information supports the decision of reintroducing the guanaco in QCNP, some aspects of this reintroduction process, such as the release method, do not always produce the expected results in terms of survival [28].

Considering that this project is a milestone in the history of reintroduction of wild species in the national system of protected areas in Argentina, the principal objective of the present work was to evaluate the current probability of persistence of guanacos in QCNP under different future supplementation scenarios, and the secondary objective was to establish management priorities to achieve the effective recovery of the guanacos in the high mountains of central Argentina.

## Materials and Methods

Delegación Regional Centro of the Administración de Parques Nacionales of Argentina grant the permission to conduct the research in the Quebrada del Condorito National Park.

### Study area

**Fig 1:**
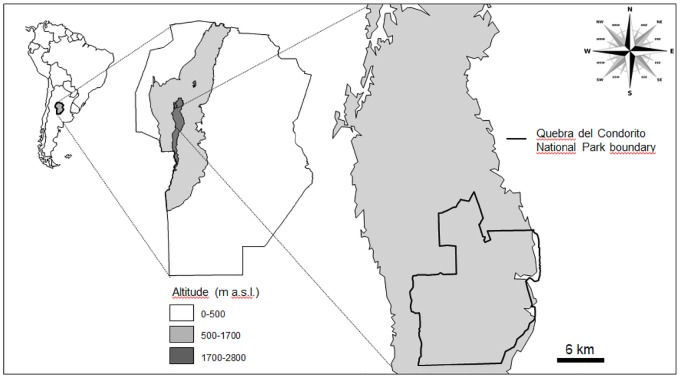
Location of Quebrada del Condorito National Park, in the high mountains of central Argentina.

QCNP is located in the upper portion of the Córdoba mountains (1700–2800 m a. s. l., 31°34’S, 64°50’W) in central Argentina, covering an area of 24 774 ha (Fig 1). The ecosystem includes grasslands and Polylepis (*Polylepis australis*) forest enclaves interspersed with rocky outcrops [29]. The climate is temperate and humid, with an annual average temperature of 8°C and rainfall of 900 mm. The area is considered a biogeographic island due to its isolation, the confluence of different streams, and the presence of diverse plant and animal species, including more than 30 endemic species [30].

### Study population

Guanacos were reintroduced following IUCN guidelines [24]. Each reintroduced guanaco was marked with a coloured and numbered plastic ear tag and a neck-band (red in males and blue in females), and nearly 30% of all released individuals were also radio-collared to facilitate subsequent monitoring. Two groups of guanacos were reintroduced in QCNP. In 2007, 113 individuals from a wild northern Patagonia population (40° 47'S, 66° 45'W) were released without being subject to a pre-adaptation period, of which 19 females and 17 males were radio- collared. Of all individuals released in 2007, only about 20% survived the first three months of the critical post-release period, with starvation and predation by puma (*Puma concolor*) being the most frequent causes of death [25]. In 2011, 25 individuals from a captive population from Buenos Aires (38°01'S, 61°40'W) were released; of these individuals, five females and two males were radio- collared. Individuals were previously subjected to a 40-day pre-adaptation period in a barnyard constructed in the Park for this purpose. More than 80% of individuals of the latter group survived the critical post-release period [28]. Nine years after the start of the guanaco reintroduction project, the population size was 24 adults and three young individuals (Table 1). Regarding causes of offspring death, of the 70 young guanacos born in QCNP since the beginning of reintroduction project, 19 individuals were preyed upon in their first months of life, 25 died during the winter period and 9 were caught in wire fences.

**Table 1:** Survival and recruitment of a reintroduced guanaco population in central Argentina (March 2007 to March 2016).

Year	N° of adult males	N° of adult males dead	N° of adult females	N° of adult females dead	N° of offspring born	N° of young that reached juvenile stage
**2008**	3	0	14	3	9	2
**2009**	4	2	12	2	9	2
**2010**	3	1	11	1	8	2
2011*	10	2	19	3	14	3
**2012**	9	2	18	2	9	2
**2013**	9	1	16	1	7	1
**2014**	8	1	16	0	6	2
**2015**	8	0	17	1	8	3

* Reintroduction of a new group of guanacos; in March 2016 the guanaco population consisted of three breeding groups (the first with one male and eight females, the second with one male and five females, and the third with one male and three females) and one group of solitary males (with five individuals).

Since 2007, the area where the guanacos were reintroduced has been monitored with two different frequencies. Between December and March, i.e., late spring-summer or rainy season, and coinciding with the guanaco's reproductive period in QCNP (which included birth, mating and first months of offspring rearing), I have visited the area once a week. From April to November, during the autumn-early winter or the dry and cold season and coinciding with the post-reproductive period, I have visited the area every two weeks, between 1000 and 1800 hours. I tracked guanacos by radio-telemetry and located them by surveying their home range on foot, on horse or by truck. Once I detected the individuals, I made focal observations with telescope and binoculars, and recorded the site coordinates with GPS. In each case, I recorded the number of individuals, sex, age group (classified as adult, juvenile and offspring), and number of the plastic ear tag, whenever possible.

### Population viability analysis

I used the stochastic population model VORTEX (http://vortex10.org/Vortex10.aspx) to evaluate extinction risk and corresponding population trend [31] of the guanaco population reintroduced in QCNP. VORTEX computes population dynamics by stepping through a series of time events and following the fate of each individual in the population. I used a standard input parameter set for most simulations (Table 2). For initial population size and structure, I used a random subset of the population between March 2008 and September 2015. Simulations were repeated 1,000 times; results were predicted over 100 years, and modified subsequently.

**Table 2:** Initial population structure and estimated mortality rates (means ± SD) used to model the reintroduced guanaco population in central Argentina.

Age (year)	Initial number of females	Female mortality (%)	Initial number of males	Male mortality (%)
0	1	76 ± 14	1	78 ± 15
1	1	18 ± 3	0	25 ± 6
2	1	5 ± 1	1	10 ± 2
> 3	12	8 ± 1	7	15 ± 3

### Population parameters

The guanaco is a social ungulate found in three basic social units: territorial family harems, non-reproductive male groups and solitary males [32]. It is a polygynous species, with dominant males maintaining their group of females by defending sites of high resource availability against other males [33], [34]. The size of a guanaco family group varies between 5 and 13 adults, with an average of 2.9 young [35]. The whole reproductive cycle of birth, mating and early lactation coincides with the best environmental conditions during and after the rainy season [36]. After 11.5 months of gestation, a female guanaco gives birth to a single offspring that is about 10% of the mother’s weight [37]. With no indication of a skewed sex ratio at birth [38], I set this parameter at parity. Predation, starvation in winter and accidents are the main causes of mortality during the first year of life, reaching values as high as 70% [39], [40]. The young stay with mothers for 1 year, with the male offspring being expelled aggressively from the adult male territory [41]. Females and males reach maturity at 2 and 3 years of age, respectively [42], [34]. I estimated mean foaling rate between 53 and 76% [38]; the fecundity period extends approximately for 14 years for males and 18 for females [43].

The data collected through continuous monitoring allowed me to follow the group composition of guanacos and their evolution over time in QCNP. Therefore, to model realistic population dynamics scenarios, I used the estimated demographic parameters for the reintroduced guanaco population: (1) specified age distribution; (2) mortality rates (percent mortality of females and males, discarding the individuals that died during the first three months of the critical post-release period, previously evaluated by [28]; (3) reproductive rates (percent adult females breeding and average number of offspring per female per year); and (4) mate monopolization (percent males in breeding pool, percent males successfully siring offspring and mean of mates/successful sire) (Tables 2 and 3). I took the remaining demographic parameters of the species (reproductive system, age of first offspring, maximum number of broods per year, mean foaling rate, and length of fecundity period) from the literature.

**Table 3:** Parameters used for modelling the reintroduced guanaco population in central Argentina.

Parameter	Description
**Mating system**	Polygynous; 38% of males and all females in the breeding pool
**Maturity**	Females at 2 years, males at 3 years
**Fecundity rate (%)**	57 (not age- or density-dependent)
**Litter size**	1 with a 1:1 sex ratio at birth
**Carrying capacity**	5802 individuals*
**Model iterations**	All scenarios repeated 1000 times and covering 100 years

* Estimated by Tavarone et al. (2007)

### Genetic considerations

Inbreeding depression according to the standard VORTEX heterosis model for mammals (accounting for 3.14 equivalents of lethal genes; [44]) did not have a significant influence on population dynamics and the corresponding extinction risk. Therefore, I did not use this variable in the model.

### Release regime

Even though the guanaco reintroduction project considered the release of one group per year from 2007 [45], only two groups were released in eight years [28]; therefore, I made the first PVA with the current population. In turn, based on previous financial and logistic limitations to maintain a continuous guanaco release regime, I assumed a realistic supplementation of 21 individuals every 4 years during a period of 12 years, with 80% of survival during translocation. In addition, I performed an optimistic scenario of release regime including a double number of individuals and age structure, but half the time between releases and for a period twice as long as the other scenario. Emigration and immigration do not occur in the study area and were not considered in the model.

### Influence of catastrophes

Two main types of local catastrophic events can occur in QCNP: fires and food shortage, leading to starvation during harsh winters, which were modelled at different probabilities of effect on reproduction and mortality (Table 4). Fires (either intentional or naturally caused by lightning) affected almost 20% of the study area between 1999 and 2011. Although most of them are extinguished by fire-fighters before they reach large extensions, in QCNP particularly severe fires occur approximately every six years [46], for example, the last fire occurred in august 2015 and affected nearly 40% of the Park. Nonetheless, fires are considered to have a low impact on reproduction and mortality due to the guanaco’s capacity to escape and the rapid regrowth of pastures. Furthermore, there is evidence that the main driver of habitat selection by reintroduced guanacos in the mountain rangeland of central Argentina is the availability of forage of high nutrition value, which is reduced under the arid conditions of the winter season [26]. Therefore, it is assumed that most severe winter climatic conditions, which occur approximately every nine years [47], may have a slightly greater impact on the reintroduced guanaco population than fires. Nevertheless, due to model restrictions and the lack of specific information, these variables could not be modelled by age- or sex-specific characteristics, and I assumed that environmental effects on mortality and reproduction act concordantly.

**Table 4:** Probability of occurrence of natural catastrophes considered for modelling the reintroduced guanaco population in central Argentina. Severity levels are expressed as reduction factors (e.g., a severity level of 1means no influence and a severity level of 0.25 stands for the greatest influence).

Type	Frequency	Effect on reproduction	Effect on mortality
**Fires**	1/6 year	0.95	0.90
**Harsh winter**	1/9 year	0.75	0.80

### Carrying capacity

Carrying capacity was estimated at 5,802 individuals in an area of 16,509 ha, based on a pre-feasibility study of guanaco reintroduction in QCNP conducted by [45], at the request of the NPA. Because the present population size is far smaller than the estimated carrying capacity, the influence of this parameter on the model results was limited.

## Results

**Fig 2:**
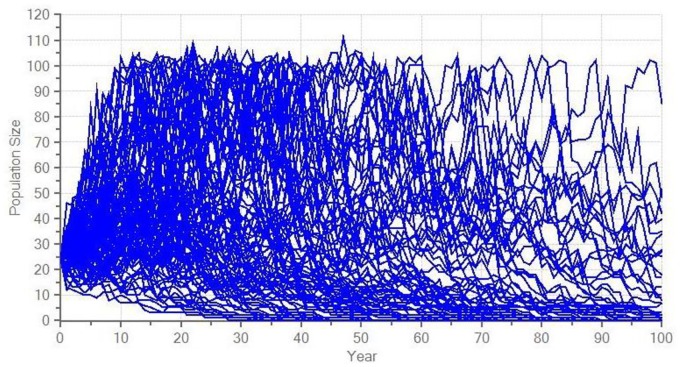
Modelled stochastic fluctuations for the current reintroduced guanaco population in central Argentina.

**Fig 3:**
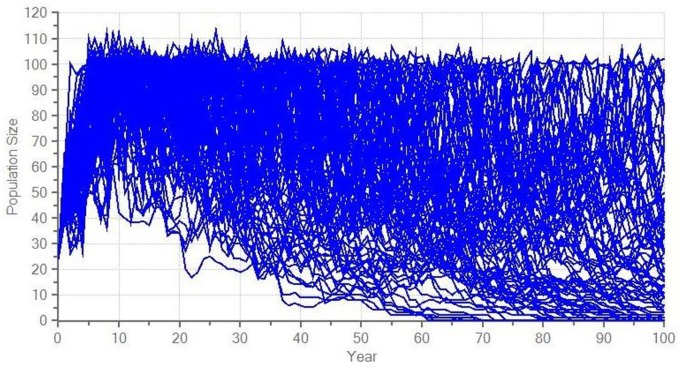
Modelled stochastic fluctuations for a realistic scenario on the reintroduced guanaco population in central Argentina.

**Fig 4:**
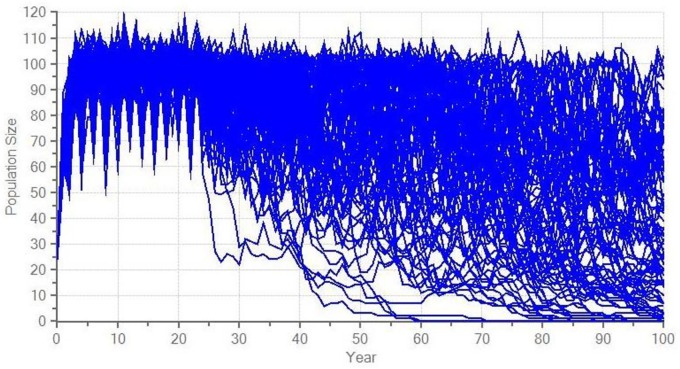
Modelled stochastic fluctuations for an optimistic scenario on the reintroduced guanaco population in central Argentina.

The model suggests a high probability of extinction for the current reintroduced guanaco population (Fig 2), whereas supplementation scenarios provide a positive growth rate, although it is not significantly different from 0 (Table 5). A realistic supplementation scenario reduces the risk of extinction by half and only an optimistic scenario guarantees more than 75% of surviving probability over the next 100 years (Figs 3 and 4).

**Table 5:** Growth rate and extinction risk (means ± SD) cover 100 years of three population viability analysis scenarios for reintroduced guanacos in central Argentina. Growth rates are specified without truncation by carrying capacity.

Description Scenario	Growth rate	Probability of extinction (%)	Mean time to first extinction	Final population size
**Current population**	-0.029 ± 0.198	79.1 ± 1.9	60 ± 22	5 ± 14
**Realistic supplementation**	0.012 ± 0.202	36.4 ± 0.9	82 ± 12	39 ± 29
**Optimistic supplementation**	0.065 ± 0.244	22.3 ± 0.5	96 ± 14	53 ± 28

To evaluate the fate of the released population in the long term, I used the probability of extinction after 100 years as an indicator of potential success. Assuming the same parameters and catastrophe levels, an initial population of >134 guanacos would be necessary to achieve a 99% probability of survival over 100 years.

## Discussion

The model predicts that the current reintroduced guanaco population in the upper belt of central Argentina could be extinct in the next decades. Although variation within the models limits their predictive value [48], the field data recorded by monitoring conducted in the last eight years indicate a poor growth capacity of the established guanaco population. The number and frequency of released animals also show a strong effect on risk extinction, with increasing frequency and number of animals released reducing that risk. Accordingly, the modelled scenarios show that the continuity of the expected supplementation increases the probability that the population persists during the next 100 years, and that if such supplementation were more frequent and with a larger number of individuals, the possibility of success of the project would be greater.

Nevertheless, even though supplementation has proved to be a powerful management strategy for wildlife reintroduction projects [4], [5], [13], [14], the model also shows that, even when supplementation were doubled, the probability of extinction would not reach the desired value below 5%. One possible reason for this result is that in gregarious animals, density dependence processes increase the probability of extinction [49]. Given the gregariousness characteristic of the species [32], if preformed reproductive groups were released, it can be assumed that this management practice would play an important role in the population dynamics of the reintroduced guanacos. Furthermore, even though natural catastrophes cannot be addressed directly by individual management actions, other strategies, such as increasing the pre-adaptation period or removing fences, can reduce the mortality of individuals in QCNP [28].

The causes of local extinction of the guanaco in the study area have been removed because rangers actively control poaching in the Park. Furthermore, guanaco population density seems to be independent of habitat structure or predation risk [50], [51], whereas the observed recruitment values are within the range recorded for the species [39–42], However, predation by puma could be the main cause limiting growth rate of this reintroduced guanaco population, given the low number of births since the first release of individuals and the higher density of pumas in the QCNP than in neighbouring areas [52]. This particular aspect should be taken into account in future reinforcement strategies, and before release, new guanaco reproductive groups could be maintained in the pre-adaptation crowd pen constructed in the Park for a minimum of three months after birth.

The guanaco reintroduction project in QCNP was developed following the IUCN guidelines for reintroductions, with continuous monitoring and the performance of the required pre- and post- release studies. Nevertheless, although in the original draft successive releases over the following years were planned, they could not be executed until 2011. At present the need of reinforcement of the reintroduced guanaco population has been included in the updated management plan of the QCNP. In turn, some management decisions, such as the absence of a pre-adaptation period in the first released groups, reduced the total number of established individuals [28]. Nevertheless, different studies conducted on the guanaco population reintroduced in QCNP indicated that the individuals that survive the critical post-release stage were adapted in terms of behaviour [25], habitat selection [26], and diet [27]. Preliminary evidence shows that the guanacos could contribute to the ecological restoration of the area (unpublished data), reinforcing the importance of this project focused on restoring natural processes rather than addressing only extinction risk [53].

The survival and fecundity rates estimated during the eight years of the guanaco reintroduction project in QCN were similar to those of other wild populations that show, in general, a high adult survival and low recruitment rate [32], [33], [40], [54–56]. Therefore, the high extinction probability of the current reintroduced guanaco population would not be related to reproductive success or population structure, but to the small number of individuals, which increases the vulnerability to suffer negative effects of stochastic dynamics [57], [58]. Even though substantial progress in reversing defaunation is being achieved around the world, some projects have still not reached the ultimate success [4], [5], being also strongly biased towards Europe and North America [3]. The guanaco reintroduction project in the upper belt of the mountains of central Argentina can be considered to have been partially successful since its start in 2007, because some reproductive groups have been established during the last eight years. However, based on the results of the present PVA, the project could fail if population reinforcements do not continue in the next decades until a minimum population size is ensured so as to achieve a 99% probability of persistence for at least the next 100 years. Constant supplementation of individuals is necessary to increase the chances of long-term survival for this guanaco population reintroduced in QCNP. Therefore, the logistic and structural constraints that have reduced the frequency of the release regime in the last years should be overcome by the NPA of Argentina; otherwise, previous efforts will be lost.

In wildlife reintroduction projects, building decision frameworks is both a socio-political and a scientific process [16], and particularly when future funding is uncertain, two objectives need to be met: maximizing population size after release and minimizing the degree to which the population falls below some threshold [59]. Accordingly, if appropriate management measures are taken, the persistence probability of the guanaco population reintroduced in QCNP can be maximized. Likewise, continuous monitoring of guanaco population parameters and subsequent modelling exercises are needed, as an adaptive management tool to assess the long-term success or failure of this reintroduction project, and therefore to contribute with the ecological restoration of the upper belt of the mountains of central Argentina.

## Supporting Information

S1 Table Extinction risk for current population scenario. (TXT)

S2 Table Extinction risk for realistic supplementation scenario. (TXT)

S3 Table Extinction risk for optimistic supplementation scenario. (TXT)
